# Acknowledgement to Reviewers of *Cells* in 2018

**DOI:** 10.3390/cells8010060

**Published:** 2019-01-16

**Authors:** Cells Editorial Office

**Affiliations:** MDPI, St. Alban-Anlage 66, 4052 Basel, Switzerland

Rigorous peer-review is the corner-stone of high-quality academic publishing. The editorial team greatly appreciates the reviewers who contributed their knowledge and expertise to the journal’s editorial process over the past 12 months. In 2018, a total of 288 papers were published in the journal, with a median time to first decision of 14.5 days and a median time to publication of 35 days. The editors would like to express their sincere gratitude to the following reviewers for their cooperation and dedication in 2018:

Abdalla, Maher Y.Li, YingcuiAdamek, AgnieszkaLi, ZhiyuAdrain, ColinLiao, Albert T.Ai, YongLiao, Yi-JenAla, UgoLiby, KarenAlachkar, HoudaLiedtke, WolfgangAldskogius, HåkanLiehr, ThomasAlexeyev, MikhailLightfoot, AdamAlfano, DanielaLim, Jae HyangAlloza, IraideLin, Pei-HuiAlpizar, Yeranddy A.Lin, YiguangAlvarado-Kristensson, MariaLin, Ying-HungAmpofo, EmmanuelLin, YongAnandhan, AnnaduraiLindholm, DanAndre, RalphLindsay, Andrew J.Andreassi, MariaLindström, Mikael S.Anifandis, GeorgeLisby, MichaelAnnesley, SarahLiu, BeidongAnsari, RaisLiu, YieAntcliffe, DavidLiu, YongqingAnthias, ChloeLiu, ZiqingAran, GemmaLiu-Smith, FengArany, PraveenLombardi, AngelaArguelles, SandroLopes, SusanaArndt, Patrick G.López, Manuel C.Arnoult, NausicaLoré, KarinArtemenko, YuliaLorencini, MarcioAskjaer, PeterLow, PeterAspenström, PontusLu, QuanlongAunapuu, MarinaLu, ZhanpingAvignone, ElenaLuce-Fedrow, AlisonAyers, DuncanLüders, JensAzizi, EbrahimLui, Edmund Man KingBabenko, Nataliya A.Lukyanov, KonstantinBaer, Patrick C.Luna, Jesus IsaacBailer, Susanne M.Lundqvist, AndreasBaker, MatthewLuo, YonglunBaldwin, Albert S.Luthra, AmitBalestrieri, EmanuelaLuxton, GW GantBandres-Ciga, SaraMachado, RicardoBanerjee, AditiMachlus, KellieBanerjee, KalpitaMacParland, SonyaBanti, Christina N.Maher, PamelaBarabas, PeterMajima, Hideyuki J.Barata, JoãoMajor, IanBarbie, DavidMakarevich, PavelBarbolina, MariaMakarevitch, IrinaBarry, GuyMakishima, MakotoBartulos, OscarMakiyama, TakeruBasak, SujitMalara, AlessandroBaserga, Susan J.Malemud, CharlesBasoli, ValentinaMalemud, Charles J.Basu, DipanjanMalpeli, GiorgioBattino, MaurizioMangala Prasad, VidyaBattistelli, CeciliaMannell, HannaBattistini, LuciaMao, MengBaudin, BrunoMarcal, HelderBawadekar, MandarMarcel, VirginieBecker, GuillaumeMarchi, SaverioBecker, ThomasMarín, MercedesBeitz, EricMarinov, GeorgiBelrose, Jillian CorinneMarkert, ElkeBełtowski, JerzyMarnetto, FabianaBender, DavidMartens, GerardBenjamin, EngelMartikainen, RiikkaBergdahl, AndreasMartín-Antonio, BeatrizBerkhout, BenMartínez De Paz, Pedro JoséBerry, DanielMartínez-Contreras, Rebeca D.Berta, TemuginMartínez-Soler, FinaBiagini, GiuseppeMartins, IsabelBielak-Żmijewska, AnnaMaruyama, IchiroBierhoff, HolgerMastro, AndreaBilyachenko, Alexey N.Matsumoto, TakuyaBisulli, FrancescaMattei, CesarBiswas, SangitaMcCown, Thomas J.Boehme, Karen A.McDermott, GerryBogusławska, JoannaMcMillin, MatthewBohnert, MariaMegraw, TimothyBon, RobinMegyeri, KlaraBorger, PieterMei, Ya-FangBorghini, AndreaMeinke, PeterBosch, Megan E.Mendez-Sanchez, NahunBousquet, CorinneMenon, ManojBoyer-Guittaut, MichaëlMerdes, AndreasBozzaro, SalvatoreMetzinger, LaurentBrachet, EtienneMeunier, AliceBrand-Saberi, BeateMichels, Alexander J.Brandt, CurtisMichiels, CarineBraun, Ralf J.Mielcarek, MichalBreunig, JoshuaMiele, Adriana EricaBrevern, AlexandreMilavetz, BarryBrewster, JayMiletta, MariaBriata, PaolaMillar, J. CameronBrizuela, LeyreMiller, Robert H.Broer, StefanMinami, YoichiBrown, Christine E.Mirete, SalvadorBrüstle, AnneMishra, AbheepsaBrzóska, EdytaMishra, VivekBugla-Płoskońska, GabrielaMitchell, Brian J.Bugliani, MarcoMitchell, David A.Bui, Jack D.Miyagoe-Suzuki, YukoBumah, VioletMlera, LuwanikaBürglin, Thomas R.Modhiran, NaphakBurke, RichardMonsalve Pérez, Maria P.Burman, Jonathon L.Montagne, KevinBurstyn-Cohen, TalMorales, JulioButt, GrantMorel, EtienneBzducha-Wróbel, AnnaMorgan, Michael A. M.Cai, ZhengxinMoriguchi, TakayaCalì, TitoMoriwaki, KentaCalvo Polanco, MonicaMorsch, MarcoCampello, SilviaMouzaki, AthanasiaCarapelli, AntonioMuimo, RichmondCardile, VeneraMüller-Taubenberger, AnnetteCardullo, RichardMünsterberg, AndreaCariati, FedericaMurakami, YasufumiCascione, MariafrancescaMuraki, KatsuhikoCaspari, ThomasMurooka, ThomasCassinelli, GiulianaMurray Stewart, TracyCastresana, JavierMusumeci, GiuseppeCeccarelli, MatteoMuzio, MartaCelli, AnnaMyre, MichaelCeresa, BrianNagata, EiichiroChakraborty, AnirbanNair, ShinyChalah, Moussa AntoineNakagawa, TakayukiChang, Hao-XunNakagawa, TakuroChang, Hsueh-WeiNakamura, NobuhiroChang, Kee-LungNakamura, TsukasaChatterjee, IshitaNakanishi, AkiraChaves-Pozo, ElenaNakatsukasa, KunioChen, Guang-ChaoNalluri, JosephChen, Hsin-ChienNanba, DaisukeChen, Kok HaoNandi, SaikatChen, Szu-YuanNarayanan, AnandChen, Wei-ShengNarita, AkihiroChen, YanaNasser, MohdChen, Yu-ChihNavedo, Manuel F.Cheng, DeNawaz, MuhammadCheng, HailiNaya, FranciscoCheng, Juei-TangNayak, TapanCheng, Yu-ShanNegishi, TakayukiCheon, Yong-PilNemoto, KiyomitsuCherry, JonathanNepal, MadhavCheshenko, NataliaNeureiter, DanielChevillard, ChristopheNeuzillet, CindyChitraju, ChandramohanNew, ElizabethChlanda, PetrNewsome, Timothy P.Cho, Ssang-GooNievergelt, Adrian PascalChoo, Byung KilNigris, Filomena DeChou, Cheng-YangNikolakopoulou, Angeliki M.Chou, Jyh-HorngNiland, StephanChoudhary, SanjeevNilsen-Hamilton, MaritChrist, BrunoNolan, KarenChristensen, AlanNoubissi, Felicite KamdemChueh, Pin JuNüssler, Andreas K.Chun, Kyung-SooNyström, AlexanderChung, Man-KyoObara-Michlewska, MartaCiarrocchi, AlessiaObermayr, EvaCicciù, MarcoO’Connell, Kevin F.Cimini, DanielaO’Day, DantonCiuffreda, LudovicaOfir, RivkaColanzi, AntoninoOhashi, NaroColeman, MichaelOhe, KenjiColeman, PaulOhta, YukoComincini, SergioOkada, TomoyoCommichau, Fabian M.Oliva, JoanCommisso, MauroOliva, RominaCondello, MariaOlson, MichaelContestabile, RobertoOmar, AhmadConti, HeatherOng, QunxiangConway, Daniel E.Opattova, AlenaCorbet, CyrilOrgel, JosephCordenonsi, MichelangeloOriola, DavidCortese, KatiaOrr, BernardoCorvigno, SaraOry, StéphaneCotman, SusanOskeritzian, Carole A.Coulon, StéphaneOtt, MartinCourtois, GillesPackiam-Dayalan, AntonyCourty, JoséPaeshuyse, JanCowman, MaryPaggetti, JérômeCozzi, CristinaPal, KasturiCrawford, LindseyPalma Sircili, MarceloCrawley, Angela M.Pangestu, MulyotoCrompton, TessaPaolillo, MayraCummins, EoinPapa, SimonettaCusanelli, EmilioPapaccio, GianpaoloDaigaku, YasukazuParadowska-Gorycka, AgnieszkaDakhlallah, DuaaPark, Sung-GyooDal Ben, MatteoPark, Tae HwanDalhaimer, PaulParker, Catherine C.Dan, Han C.Parry, GeraintDarlington, PeterPaszek, PawelDas, AmitavaPathipati, PraneetiDas, ViswanathPawelek, JohnDasgupta, SantanuPayer, BernhardDash, BirajaPedersen, Per AmstrupDave, SandeepPellicioli, AchilleDe Almeida, AndreiaPeng, TengDe Filippis, Elena AnnaPentieva, KristinaDe Lago, EvaPeppelenbosch, MaikelDe Petrocellis, LucianoPereira, Cláudia Maria F.De Rivero Vaccari, Juan PabloPereira, LuisaDe Toni, LucaPeretto, Paolo MarcelloDealy, Caroline N.Perrais, MichaëlDeben, ChristophePetersen, BjörnDegrassi, FrancescaPetit, Patrice X.Dellambra, ElenaPezzella, FrancescoDeng, Win-PingPhatarpekar, PrasadDenko, NicholasPickett, HildaDharmawardhane, SuranganiePierzchalski, PiotrDhungel, BijayPinar, AnitaDi Donato, MarziaPinho, DianaDi Fazio, PietroPinto, MilenaDi Liegro, ItaliaPiosik, JacekDi Nardo, AnnaPiprek, Rafal P.Di Paola, RosannaPirity, Melinda K.Didonna, AlessandroPittalà, ValeriaDiefenbach, Russell J.Piva, TerrenceDietrich, AlexanderPlatt, Jeffrey L.Dijkstra, Johannes M.Platta, HaraldDiMauro, SalvatorePocock, JenniferDing, Wen-XingPoggi, AlessandroDiógenes, Maria JoséPoggio, PaoloDittmar, ThomasPohl, ChristianDityatev, AlexanderPohl, PeterDonninger, HowardPolakovičová, MájaDoucoure, SouleymanePolishchuk, RomanDoye, ValériePonnambalam, SreenivasanDráber, PavelPoole, DanielDransfield, IanPope, ChadDuan, ZhijunPort, Sarah A.Dudas, JozsefPossik, EliteDunbar, Gary L.Pountney, DeanDuncan, Robert KeithPowell, Timothy R.Dutta, ArijitPrashar, SanjivDziegiel, PiotrPreto, AnaEar, JasonPrevost, GillesEarp, H. SheltonProctor, CaroleEden, EmilyProhaska, RainerEder, PiotrPu, JingEggleton, PaulPuchkova, Ludmila V.Ehrnhoefer, DagmarPulinilkunnil, ThomasEid, NabilQian, GuoqingEleni, AnastasiadouRadošević-Stašić, BiserkaEngeland, Christine E.Radwan, JacekEremeeva, MarinaRahe, Michael C.Escher, PascalRahman, Masmudur M.Espinosa-Diez, CristinaRaimondo, StefaniaEtienne-Manneville, SandrineRamaiahgari, Sreenivasa C.Eyo, UkpongRamalingam, NagendranFabris, LindaRamaraj, PanduranganFaehling, MartinRamsköld, DanielFakhouri, Walid D.Ranieri, DaniloFan, Guo-ChangRaspanti, MarioFang, JunnanRavelonandro, MichelFang, XiaolanRay, RanjitFantini, MassimoRazumilava, NataliyaFasler-Kan, ElizavetaRebelo, SandraFerrante, ClaudioReddy, SakamuriFerrio, Juan PedroRedondo-Muñoz, JavierFilipeanu, CatalinReeve, AmyFilipek, AnnaReginato, Mauricio J.Fillmore, HelenReilly, SvetlanaFimia, Gian MariaResch, UlrikeFiszer, AgnieszkaRiar, Amanjot KaurFlamion, BrunoRibot, JoanFletcher, Nicola F.Richly, HolgerFolco, Hernan DiegoRilla, KirsiFölsch, HeikeRiobo-Del Galdo, NataliaFonseca, BrunoRizzo, MilenaForini, Francesca S.Roberts, Stephen K.Forrest, MarcRocha, SoniaFouquerel, EliseRocha-Ferreira, EridanFranco, DiegoRodrigo, LuisFrémont, StéphaneRodrigo, Sara MoralesFreytes, DonaldRodríguez, AmaiaFrič, JanRodriguez-Cueto, CarmenFrischauf, IreneRogakou, EmmyFröhlich, Leopold F.Rogozhin, EugeneFrossard, Jean-PierreRomero-Lorca, AliciaFujii, TomomiRonai, Ze’ev A.Fujiki, YukioRoos, Wynand PFulzele, SadanandRosenberger, Thad A.Furukawa, TatsuhikoRosende, Eugenia GonzálezFurusawa, YukihiroRossignol, JulienGadalla, Shahinaz M.Royo, FelixGagliano, NicolettaRubart, MichaelGagliano, TeresaRugarli, Elena I.Gajdács, MárióRusmini, PaolaGaletti, MariclaRusso, AnnapinaGaligniana, Mario D.Russo, AntonellaGalileo, Deni S.Russo, Gian LuigiGall Troselj, KoraljkaRusso, Matteo A.Gallay, PhilippeRusso, ValentinaGalvez, Eva M.Rzepecki, RyszardGanea, DoinaSá, RosáliaGantenbein, BenjaminSaba, Nakhle S.Garinis, GeorgeSabirov, Ravshan Z.Gauthier-Rouvier, CecileSacconi, LeonardoGavara, NúriaSadkowski, TomaszGentile, FabrizioSaint-Pol, JulienGeorge, VinojSaito, TamaoGeorgiev, VasilSaitoh, KenjiGericke, MartinSakai, SatoshiGerlitz, GabiSakurai, HidetoshiGharaibeh, BurhanSalgar, ShashikumarGhislat, GhitaSamuels, IvyGiacca, MauroSanak, MarekGiakountis, AntonisSanchez-Alcazar, JoseGiannakouros, ThomasSánchez-Madrid, FranciscoGibbs, Bernhard F.Sander, VeronikaGigli-Bisceglia, NoraSantarpia, LiberoGil, MinchanSantos, José MariaGinhoux, FlorentSantulli, GaetanoGiovannoni, RobertoSarraf, ShireenGisterå, AntonSaulnier-Blache, J. S.Giuliano, CallainiSavoldo, BarbaraGiusti, IlariaSayer, JohnGłąbska, DominikaSchenone, SilviaGligorijevic, BojanaSchirmer, EricGoberdhan, Deborah C. I.Schmid, Johannes A.Goel, PeeyushSchmidtke, GunterGoglia, FernandoSchmitz, John C.Goldberg, AlfredScholz, Carsten C.Goldhawk, Donna E.Schönthal, Axel H.Goldsmith, Jason R.Scott, CharlotteGolenhofen, NikolaSedmikova, MarketaGómez-Pastor, RocíoSellner, JohannGonzález-González, RogelioSenpuku, HidenobuGorbatyuk, Oleg S.Serino, GiovannaGorska-Ponikowska, MagdalenaSgodda, MalteGosselet, FabienShabanpoor, FazelGowran, AoifeShanker, AnilGozzelino, RaffaellaSharma, GeetanjaliGrãos, MárioSher, Yuh-PyngGreene, CatherineShibuya, HiroshiGreenwood, Michael T.Shim, Joon W.Gregor, IngoShin, Joo-HoGrigorieva, Elvira V.Shin, Vivian YvonneGris, DenisShiraishi, TakehikoGroenendyk, JodyShityakov, SergeyGroschner, KlausSiddiqui, ImranGroßhans, JörgSiegmund, BrittaGrunig, GabrieleSies, HelmutGruss, OliverSilvan, UnaiGu, YianSimanshu, Dhirendra K.Guérineau, NathalieSimard, MarcGuerreiro, Joana F.Šimoník, OndřejGuha, SoniaSingh, AnirudhaGuichard, PaulSinha, DebottamGuinamard, RomainSkovsted, Gry FrejaGulbins, ErichSlawson, ChadGundamaraju, RohitŚmieszek, AgnieszkaGuo, ChuanyongSobue, KenjiGuo, FenghaiSochaj-Gregorczyk, Alicja MariaGuo, Zong ShengSokoloski, KevinGurevich, Vsevolod V.Solaini, GiancarloGurrapu, SreeharshaSomma, PatriziaHafizi, SassanSong, ChunhuaHak, SjoerdSong, JianxunHancock, JohnSosna, JustynaHao, YajingSousounis, KonstantinosHara-Chikuma, MarikoSoveral, GraçaHärkönen, PirkkoSpang, ChristophHarper, JamesSprio, Andrea ElioHarris, Andrew R.Spyridopoulos, IoakimHarvey, AdamSrivastava, AkhilHata, YutakaStaal, JensHe, PeijianStallforth, PierreHeeger, Peter S.Starr, DanielHegmann, EldaStary, Creed MichaelHeinbockel, ThomasStathopulos, PeterHemley, Sarah J.Steger, GertrudHenry, Ryan A.Stellato, FrancescoHerrero, EnriqueSteward, RobertHerrmann, Harald O.Stewart, JasonHieda, MikiStilli, DonatellaHildt, EberhardStirling, PeterHinds Jr., Terry D.Stixova, LenkaHirabayashi, YusukeStrudwick, XantheHirata, YokoSuarez-Bregua, PaulaHissa, BarbaraSubedi, DineshHlavaty, JurajSugasawa, KaoruHo, Yuan-SoonSühs, Kurt-WolframHodgkinson, ConradSukseree, SupawadeeHolaska, James M.Sullivan, ConHoogenraad, Nicholas J.Suman, ShubhankarHöpken, Uta E.Surguchov, AndreiHorhat, Florin GeorgeSuzuki, AussieHorner, AndreasSydor, SvenjaHowe, Alan K.Tackenberg, ChristianHozumi, KentaroTaguchi, Y-h.Hsia, Shih-MinTai, AndrewHsuan, JustinTajiri, NaokiHu, ZhengqingTakahashi, ToshioHuang, Hsuan-YingTakei, KohtaroHuang, Yu-ChuenTanaka, MasayoshiHuber, RobertTanaka, NobuyukiHuwiler, AndreaTang, Bor LuenHwang, Pung PungTang, QiyiIannuzzi, ClaraTatebe, HisashiIchikawa, HiroshiTeixeira Tomaz, CândidaIglesias-Linares, AlejandroTeixeira, Fábio G.Ikeda, MasahiroTempfer, HerbertImig, JohnTerradas, MarionaImlach, WendyTerzaghi, William BryanIncitti, TaniaTeschke, RolfIneichen, BenjaminTezil, TugsanIsemura, MamoruThackray, AlanaIshitsuka, YoichiThammina, ChandraIto, TakashiThéry, ManuelItoh, YoshifumiThomson, David M.Iwamoto, ShotaroThyagarajan, BaskaranIzawa, TakeshiTikkanen, RitvaIzumchenko, EvgenyTilly, JonathanJablonska, EwaTitorenko, VladimirJames, Nicholas G.Toietta, GabrieleJanas, TadeuszToivanen, RoxanneJanikowska, GrażynaTolosa, EzequielJensen, Lars HenrikTomar, DhanendraJeppe, EmmersenTong, ManJewett, AnahidTongaonkar, PrasadJia, DongyaTönges, LarsJiang, SizunToret, Christopher P.Jiao, ChenTorre, Maria LuisaJohansson, StaffanTrant, JohnJohnson, Jason L.Trapani, ValentinaJones, Donna C.Trépout, SylvainJones, Kathryn MarieTrollope, AlexandraJonscher, Karen R.Tsujita, TadayukiJonsson, Anna HelenaTsukahara, ToshifumiJordan, PeterTsukiyama-Kohara, KyokoJosef, KapfhammerTuorto, FrancescaJuarranz, YasminaTups, AlexanderJung, ChristianTurley, Eva A.Kaeffer, BertrandTuttolomondo, AntoninoKaitsuka, TakuUchio, YujiKale, AbhijitUebe, RenéKalogianni, DespinaUmar, Akhilesh K.Kaltschmidt, BarbaraUrbanelli, LorenaKaltschmidt, ChristianUsui, MichihikoKanazawa, MasatoUygun, SahraKang, Hee Y.Uziel, OritKang, LiVaandrager, Arie B.Kang, Tong HoVáclavíková, RadkaKango-Singh, MadhuriVainio, SeppoKao, Shu-HueiValenti, Maria TeresaKasai, FumioVan Der Watt, PaulineKathuria, HimanshuVan Houten, BenKatsuhara, MakiVan Noorden, CornelisKauppinen, AnuVechetti, IvanKe, BiboVeenman, LeoKeller, AndreasVeeramani, SureshKelley, EricVega, Francisco M.Keniry, MeganVergara, DanieleKenneth, NiallVernetti, Lawrence A.Ketkar, AmitVersino, ElisabettaKhacho, MireilleVezzoni, PaoloKhatri, BhuwanVincentini, OlimpiaKheradpezhouh, EhsanVinnedge, Lisa PrivetteKhoo, Bee LuanVivacqua, AdeleKido, Mizuho A.Vivo, MariaKierkus, JaroslawVladimirov, Vladimir I.Kietzmann, ThomasVlashi, ErinaKim, Hyung SikVogelaar, Christina FranciscaKim, Jeong-RaeVon Knethen, AndreasKim, Jin-WookVon Laer, DorotheeKim, Kyung-SuVulliamy, ThomasKim, Sang-WeWakao, MasahiroKimata, YukioWan, YinshengKindy, MarkWang, Bang-AnKing, JasonWang, KaiKirberger, MichaelWang, ShyiwuKitanaka, AkiraWang, YuqiKlugbauer, NorbertWang, ZhenKlupp, BarbaraWaraky, AhmedKnoll, MarkoWarren, SeanKo, Jane L.Watson, LaurenKo, Shun‑YaoWedge, SteveKobayashi, SatoruWeigand, AnnikaKobayashi, YoshiroWildemann, BrittKoivisto, AriWillcox, MarkKolios, GeorgeWingert, RebeccaKölling-Paternoga, RalfWinuthayanon, WipaweeKononenko, NataliaWłoga, DorotaKoonce, MichaelWoitalla, DirkKorac, AleksandraWojtyla, ŁukaszKoska, JurajWöllert, TorstenKotsikorou, EvangeliaWong, Fung-FuhKotula-Balak, MalgorzataWong, Ka-ChunKotyla, Przemyslaw J.Wong, Melissa H.Kovacic, HervéWong, Richard W.Koyama, TomotsuguWoods, DoriKozak, AshotWu, Chi-ReiKozłowski, HenrykXiang, XinKrajewska-Włodarczyk, MagdalenaXiao, XiangweiKretz-Remy, CaroleXiaowu, ChenKrishnakumar, ShyamXie, WankunKubaczka, CarolineYAĞCI, TamerKubatka, PeterYager, EricKucerova, LuciaYamakoshi, KimiKudoyarova, GuzelYamamoto, AyumuKuehl, MichaelYamanouchi, KeitaroKues, Wilfried A.Yamauchi, YoheiKuhn, WilfriedYan, ShengminKujan, OmarYang, KunKumamoto, EiichiYao, JiayiKumar, BinodYe, HaobinKumari, AshaYe, WenleiKunz, WolframYeo, SynKurdowska, Anna K.Yeung, Tsz-LunKusuma, GinaYool, AndreaKwon, Tae-HwanYoshida, HiderouLahiri, AmitabhaYou, SeungkwonLamarche-Vane, NathalieYu, Chia-LiLamas, José RamónYu, HaojieLancel, SteveYuan, XueLanning, NathanYung, Hong-WaLaPensee, ChristopherZaehres, HolmLarrosa, MarZahid, MalihaLasagni, LauraZakrzewska, MalgorzataLasfar, AhmedZamyatnin, Jr., Andrey A.Lassot, IrénaZaphiropoulos, PeterLattanzi, GiovannaZarkovic, NevenLau, Kin-MangZeilinger, CarstenLaurencin, Cato T.Zhan, LingLauth, MatthiasZhang, DeliangLazarou, MichaelZhang, DuoLebaron, RichardZhang, KeLederkremer, Gerardo Z.Zhang, LeiLee, Chang HoonZhang, ShulingLee, GabsangZhang, Xiao-BoLee, Hsin-ChenZhang, YunkaiLee, Jun HeeZhao, XiaoaiLee, Myung-ShinZheng, XintingLee, TaesupZhong, CuiqingLeiphrakpam, Premila D.Zhou, BangjunLemmens-Gruber, RosaZhou, BeiyanLéon, CatherineZhou, Carol L. EcaleLesage, SuzanneZhou, ChangchengLeube, RudolfZhou, ZhixiongLevin, David E.Zhu, Michael X.Levy, EfratZorov, Dmitry BorisovichLevy, StevenZou, ChunbinLi, QiangZuliani, Juliana PavanLi, Shengwen CalvinZupancic-Salek, SilvaLi, XiaoguangZuryn, Steven

